# Systemic predictors of symptom burden in CRSsNP: a cross-sectional comparison of three patient-reported outcome measures

**DOI:** 10.1007/s00405-026-10014-4

**Published:** 2026-01-20

**Authors:** María Alharilla Montilla-Ibáñez, Javier Modesto García-Fernández, María Pilar Gómez-Gallego, Rafael Lomas-Vega

**Affiliations:** 1 Department of Health Sciences, University of Jaén, Jaén, Spain; 2 Department of Otolaryngology, Hospital Universitario de Jaén, Jaén, Spain

**Keywords:** Chronic rhinosinusitis, CRSsNP, Quality of life, PROMs, SNOT-22, NOSE-E, CRS-PRO, Sleep apnea, Symptom predictors

## Abstract

**Objective:**

To examine the influence of sociodemographic and clinical variables on symptom burden in chronic rhinosinusitis without nasal polyps (CRSsNP), and to compare the sensitivity of three validated patient-reported outcome measures (PROMs)—NOSE-E, SNOT-22, and CRS-PRO—to these predictors. A planned sensitivity analysis was conducted to evaluate whether sleep-related item content influences associations with obstructive sleep apnea (OSA).

**Study design:**

Cross-sectional analytical study.

**Setting:**

Tertiary care hospital.

**Methods:**

Fifty adults with CRSsNP completed the NOSE-E, SNOT-22, and CRS-PRO questionnaires. Sociodemographic and clinical variables, including age, sex, BMI, smoking status, allergic rhinitis, asthma, OSA, and prior nasal surgery, were recorded. Univariate and multivariable linear regressions were used to identify independent predictors for each PROM. A predefined sleep-free analysis excluded sleep- and fatigue-related items (NOSE-E item 4; SNOT-22 items 11–14; CRS-PRO items 9–10).

**Results:**

Each PROM demonstrated a distinct predictor profile. In multivariable models, age and smoking independently predicted NOSE-E scores. Previous nasal surgery was the only independent predictor of SNOT-22 scores. Age and OSA were associated with CRS-PRO scores in the primary model; however, the OSA association disappeared in the sleep-free analysis, indicating that it was driven by sleep-related item content rather than sinonasal symptomatology. All models showed modest explanatory capacity (adjusted R² ≈ 0.10–0.19).

**Conclusion:**

Systemic and lifestyle factors exert instrument-specific influences on symptom reporting in CRSsNP. The apparent association between OSA and CRS-PRO is mediated by sleep-related items, underscoring the importance of considering domain structure when selecting and interpreting PROMs. PROM-based evaluations in CRSsNP should incorporate domain-aware interpretation and caution in patients with sleep comorbidities.

**Supplementary Information:**

The online version contains supplementary material available at 10.1007/s00405-026-10014-4.

## Introduction

Chronic rhinosinusitis without nasal polyps (CRSsNP) is a common inflammatory disorder of the paranasal sinuses and nasal mucosa, distinct from the polypoid phenotype in both immunopathology and clinical presentation. Representing approximately 70–80% of all CRS cases, CRSsNP is predominantly characterized by type 1 or mixed Th1/Th17 inflammation and a tendency toward tissue fibrosis rather than eosinophilic polyp formation [1]. Clinically, these patients often report facial pressure, purulent nasal discharge, and facial pain, whereas CRSwNP more frequently presents with pronounced nasal obstruction and anosmia due to polypoid mucosal edema [2, 3].

Despite the absence of polyps, CRSsNP can lead to a symptom burden and quality-of-life (QoL) impairment comparable to the polypoid phenotype. Many patients experience significant sleep disturbance, fatigue, activity limitation, and reduced productivity, highlighting the subjective impact of the disease beyond what is captured by endoscopy or imaging [4, 5]. For this reason, patient-reported outcome measures (PROMs) have become essential tools in the clinical evaluation of CRS. Among the most widely used and validated instruments are the Nasal Obstruction Symptom Evaluation (NOSE-E), the Sino-Nasal Outcome Test (SNOT-22), and the Chronic Rhinosinusitis Patient-Reported Outcome measure (CRS-PRO) [6–8], each capturing overlapping yet distinct symptom domains.

Although these instruments are commonly used in research and clinical practice, limited evidence exists regarding how systemic and lifestyle factors shape PROM scores specifically in CRSsNP. Obesity, smoking, allergic disease, asthma, and obstructive sleep apnea (OSA) have all been suggested as potential contributors to symptom severity, but their independent effects within this non-polypoid phenotype remain unclear and inconsistently reported [4, 9, 10]. Furthermore, methodological studies comparing how different PROMs respond to the same clinical predictors are scarce. Understanding whether these instruments similarly reflect the influence of systemic comorbidities—or whether they differ in sensitivity due to their domain structure—may help refine PROM selection and interpretation in CRSsNP [11].

To address these gaps, the present study evaluates a broad set of sociodemographic and clinical variables as potential determinants of symptom severity and QoL impairment in CRSsNP, using three validated PROMs administered within the same cohort. By examining these predictors concurrently across NOSE-E, SNOT-22, and CRS-PRO, we sought not only to identify which systemic and lifestyle factors exert the greatest influence on patient-reported burden, but also to understand whether these widely used instruments capture such influence in comparable ways. Particular attention was given to body mass index (BMI) and obstructive sleep apnea (OSA), two comorbidities with plausible mechanistic links to upper airway inflammation, sleep disturbance, and symptom perception, yet whose independent contribution to CRSsNP remains insufficiently characterized.

Because existing evidence suggests that both higher BMI and OSA may amplify subjective symptom experience in chronic upper airway disease, we anticipated that patients with these comorbidities would report higher scores across the three PROMs. At the same time, recognizing that NOSE-E, SNOT-22, and CRS-PRO differ in their domain structure—particularly regarding the weighting of nasal obstruction, global symptom burden, sleep-related concerns, and psychosocial impact—we expected that the strength of these associations might vary from one instrument to another. The study was therefore conceived with a dual purpose: first, to explore which patient characteristics independently predict symptom burden in CRSsNP; and second, to assess whether the three PROMs demonstrate similar or divergent sensitivity to these predictors. Given the modest sample size and the exploratory nature of the analysis, these expectations were framed as working hypotheses intended to guide interpretation rather than to establish definitive causal relationships.

## Materials and methods

### Study design and participants

This was a cross-sectional, analytical, and exploratory study conducted at the outpatient otolaryngology department of a tertiary care hospital. Consecutive adult patients (≥ 18 years) with a diagnosis of chronic rhinosinusitis without nasal polyps (CRSsNP) were recruited during routine clinical visits. CRSsNP was defined according to the criteria of the European Position Paper on Rhinosinusitis and Nasal Polyps 2020 (EPOS 2020): the presence of at least two cardinal symptoms (one of which nasal obstruction or nasal discharge) lasting 12 weeks or longer, in the absence of visible nasal polyps on nasal endoscopy [1].

### Data collection

Sociodemographic and clinical data were obtained through structured face-to-face interviews and review of electronic health records. The following variables were recorded: age (years), sex (male/female), weight (kg), height (cm), body mass index (BMI, kg/m²), educational level (elementary, high school, university), smoking status (current smoker vs. non-smoker), passive smoke exposure (living with smokers: yes/no), presence of allergic rhinitis, asthma, and obstructive sleep apnea (OSA), previous nasal surgery (yes/no), current surgical indication (yes/no), and animal exposure at home (yes/no).

BMI was calculated from measured height and weight during the clinic visit and treated as a continuous variable in all analyses. OSA was considered present when previously diagnosed and documented in the medical record. Allergic rhinitis, asthma, and smoking status were recorded based on medical history.

### Outcome measures

All patients completed the Spanish versions of three validated patient-reported outcome measures (PROMs) for chronic rhinosinusitis during the same clinic visit:

- The Nasal Obstruction Symptom Evaluation (NOSE-E) is a five-item scale that assesses the severity of nasal obstruction symptoms over the preceding month, with each item scored from 0 (“not a problem”) to 4 (“severe problem”). The total raw score is multiplied by 5 to yield a final score ranging from 0 to 100, with higher scores indicating more severe nasal obstruction. The Spanish version has shown good reliability and validity in sinonasal patients [6].
- The Sino-Nasal Outcome Test-22 (SNOT-22) is a 22-item questionnaire that evaluates a broad spectrum of CRS-related symptoms and their impact on daily life, including nasal obstruction, rhinorrhea, facial pain/pressure, sleep disturbance, fatigue, and emotional distress. Each item is rated on a 0–5 scale, yielding a total score from 0 to 110, where higher scores reflect worse health-related quality of life. The Spanish adaptation has been validated in CRS populations and is widely recommended by EPOS [7].
- The Chronic Rhinosinusitis Patient-Reported Outcome measure (CRS-PRO) is a 12-item instrument specifically developed for CRS, covering physical symptoms, sensory function, psychosocial impact, and functional limitations. Items are scored on a 0–4 scale, and the total score is obtained by summation, with higher values indicating greater symptom burden. The questionnaire has recently been adapted and validated for Spanish-speaking patients, demonstrating good psychometric properties [8].

### Statistical analysis

Descriptive statistics were calculated for all sociodemographic, clinical, and PROM variables. Continuous variables were summarized as means and standard deviations, and categorical variables as frequencies and percentages.

Linear regression analyses were used because the three PROMs (NOSE-E, SNOT-22 and CRS-PRO) provide continuous outcome scores, allowing estimation of the independent contribution of each predictor. The analytical strategy followed two stages. First, univariate linear regression models were performed to examine the association between each individual predictor (age, sex, BMI, smoking status, passive smoke exposure, allergic rhinitis, asthma, obstructive sleep apnea, previous nasal surgery, surgical indication, and animal exposure) and each PROM. Predictors with a p-value < 0.10 in the univariate analysis were considered for multivariable modelling, following recommendations for exploratory analyses with limited sample sizes.

Second, separate multivariable linear regression models were built for each PROM using a backward stepwise selection procedure. All predictors meeting the univariate screening criterion were entered into the initial model and sequentially removed until only variables with p-values < 0.05 remained. Categorical variables were entered as binary indicators according to their original yes/no coding. Adjusted R² was used to quantify the explanatory capacity of the final models.

Model assumptions (linearity, homoscedasticity, and approximate normality of residuals) were evaluated through visual inspection of residual plots and Q–Q plots. Multicollinearity among predictors was assessed using variance inflation factors (VIF), with no values indicating problematic collinearity.

To address the potential influence of sleep-related item content on the association between obstructive sleep apnea and PROM scores, a planned sensitivity analysis was performed. “Sleep-free” scores were created by removing predefined sleep- or fatigue-related items from each instrument (NOSE-E: item 4; SNOT-22: items 11–14; CRS-PRO: items 9–10). Univariate screening and backward multivariable modelling were repeated using these modified scores.

No a priori sample size or power calculation was performed. Given the sample size (*n* = 50) and the exploratory intent of the study, all models were interpreted as hypothesis-generating. All statistical analyses were conducted using SPSS Statistics version 27 (IBM Corp.).

### Ethical considerations

The study was conducted in accordance with the principles of the Declaration of Helsinki and applicable local regulations. The research protocol was approved by the Ethics committee of the Junta de Andalucía (Study Codec SICEIA-2024-003271). All participants received information about the study procedures and provided written informed consent prior to enrolment.

## Results

### Participant characteristics

Sociodemographic and clinical variables are summarized in Table 1. The sample showed a balanced distribution of sex and educational levels and included a broad range of BMI values. Comorbid allergic rhinitis, asthma, smoking, passive smoke exposure, and obstructive sleep apnea were present with variable frequencies across the cohort.

**Table 1:** Sociodemographic characteristics of the sample

Continuous variables	
Variable	Mean (SD)
Age (years)	43.0 (14.0)
Weight (kg)	79.0 (16.0)
Height (cm)	169.0 (10.0)
Body Mass Index (kg/m²)	27.5 (4.8)
Categorical variables	
Variable	n (%)
Male	30 (60.0%)
Female	20 (40.0%)
Elementary education	18 (36.0%)
High school	16 (32.0%)
University	16 (32.0%)
Smoker	9 (18.0%)
Non-smoker	41 (82.0%)
Passive smoke exposure: Yes	17 (34.0%)
Passive smoke exposure: No	33 (66.0%)
Sleep apnea: Yes	24 (48.0%)
Sleep apnea: No	26 (52.0%)
Allergic rhinitis: Yes	25 (50.0%)
Allergic rhinitis: No	25 (50.0%)
Asthma: Yes	12 (24.0%)
Asthma: No	38 (76.0%)
Previous nasal surgery: Yes	11 (22.0%)
Previous nasal surgery: No	39 (78.0%)
Surgical indication for CRS: Yes	20 (40.0%)
Surgical indication for CRS: No	30 (60.0%)
Living with animals: Yes	25 (51.0%)
Living with animals: No	24 (49.0%)

The distribution of PROM scores showed substantial heterogeneity. Mean (SD) scores were 11.34 (4.53) for NOSE-E, 45.18 (23.38) for SNOT-22, and 20.60 (10.22) for CRS-PRO. Sleep-free variants demonstrated lower total scores, as expected after removal of sleep-related items (Supplementary Table S1).

### Univariate analyses

Univariate linear regressions examining the association between each predictor and each PROM are presented in Tables 2, 3 and 4. Several variables showed associations below the predefined screening threshold (*p* < 0.10), although the pattern differed across PROMs. Age, smoking status, asthma, and OSA demonstrated associations with specific PROMs, while previous nasal surgery and passive smoke exposure were associated with SNOT-22 at the univariate level. These variables were retained for multivariable modelling according to the prespecified criteria.

**Table 2:** Univariate linear regressions for NOSE-E

Predictor	B	SE	95% CI	*p*
Age	−0.092	0.039	−0.171 to − 0.014	**0.023**
BMI	0.085	0.117	−0.150 to 0.320	0.463
Sex	0.587	0.992	−1.411 to 2.585	0.556
Education level	0.608	0.578	−0.559 to 1.776	0.304
Smoking	−3.676	1.353	−6.405 to − 0.948	**0.010**
Passive smoke exposure	−1.816	1.169	−4.175 to 0.543	0.128
OSA	−1.288	1.150	−3.605 to 1.029	0.277
Allergy	−1.116	1.191	−3.513 to 1.280	0.351
Asthma	−2.867	1.252	−5.394 to − 0.341	**0.027**
Previous nasal surgery	0.064	1.364	−2.690 to 2.817	0.964
Surgical indication	−1.288	1.150	−3.605 to 1.029	0.277
Animal exposure	−1.501	1.150	−3.787 to 0.784	0.195

**Table 3:** Univariate linear regressions for SNOT-22

Predictor	B	95% CI	*p*
Age	−0.117	−0.435 to 0.201	0.465
BMI	0.835	−0.472 to 2.142	0.208
Sex	6.805	−5.550 to 19.161	0.271
Education level	−0.882	−8.085 to 6.321	0.804
Smoking	−15.166	−29.272 to − 1.060	**0.036**
Passive smoke exposure	−11.674	−24.553 to 1.205	0.074
OSA	−9.011	−21.105 to 3.084	0.142
Allergy	−2.477	−14.634 to 9.681	0.682
Asthma	−13.578	−27.231 to 0.075	0.051
Previous nasal surgery	17.265	4.067 to 30.463	**0.012**
Surgical indication	−2.559	−15.649 to 10.531	0.697
Animal exposure	−13.233	−26.148 to − 0.319	**0.045**

**Table 4:** Univariate linear regressions for CRS-PRO

Predictor	B	95% CI	*p*
Age	−0.213	−0.382 to − 0.044	**0.015**
BMI	0.912	0.434 to 1.391	**< 0.001**
Sex	1.154	−2.272 to 4.580	0.501
Education level	0.722	−1.224 to 2.668	0.461
Smoking	−3.158	−6.101 to − 0.215	**0.036**
Passive smoke exposure	1.761	−0.695 to 4.217	0.159
OSA	−5.307	−8.231 to − 2.384	**< 0.001**
Allergy	−1.802	−4.358 to 0.753	0.162
Asthma	−2.253	−4.848 to 0.341	0.089
Previous nasal surgery	0.906	−1.735 to 3.547	0.494
Surgical indication	−1.833	−4.755 to 1.090	0.216
Animal exposure	−0.308	−2.905 to 2.289	0.814

### Multivariable models

The final multivariable regression models are summarized in Table 5. Across all three PROMs, the explanatory capacity of the models was modest, with adjusted R² values ranging from approximately 0.10 to 0.19.

**Table 5:** Multivariable linear regression models (final models)

PROM	Predictor	B	95% CI	*p*	Adj *R*²
NOSE-E	Age	−0.104	−0.187 to − 0.022	**0.014**	**0.186**
	Smoking	−3.55	−6.34 to − 0.77	**0.014**	
SNOT-22	Previous nasal surgery	+ 17.28	+ 3.95 to + 30.61	**0.012**	**0.106**
CRS-PRO	Age	−0.217	−0.406 to − 0.028	**0.026**	**0.168**
	OSA	−5.96	−11.31 to − 0.60	**0.030**	

For NOSE-E, age and smoking status remained independently associated with symptom severity.

For SNOT-22, previous nasal surgery was the only independent predictor.

For CRS-PRO, age and obstructive sleep apnea were retained in the final model.

Variance inflation factors for all predictors included in the final models are presented in Table 6. All variance inflation factors were close to 1.0 (range: 1.00–1.02), indicating negligible multicollinearity among predictors.

**Table 6:** Variance inflation factors (VIF)

Model	Predictor	VIF
NOSE-E	Age	1.00
	Smoking	1.00
SNOT-22	Previous nasal surgery	1.00
CRS-PRO	Age	1.02
	OSA	1.02
NOSE-E (sleep free)	Age	1.00
	Smoking	1.00
SNOT-22 (sleep free)	Previous nasal surgery	1.00
CRS-PRO (sleep free)	Age	1.00

### Sensitivity analysis: sleep-free scores

To examine whether the association between OSA and symptom burden was influenced by sleep-related questionnaire content, sleep-free PROM scores were calculated and analyzed using the same modelling strategy. Results are shown in Table 7.

**Table 7:** Sensitivity analysis using sleep-free scores

Sleep-free PROM	Predictor	B	95% CI	*p*	Adj *R*²
NOSE-E	Age	−0.094	−0.159 to − 0.029	**0.005**	**0.189**
	Smoking	−2.42	−4.62 to − 0.22	**0.032**	
SNOT-22	Previous nasal surgery	+ 14.36	+ 3.66 to + 25.06	**0.009**	**0.114**
CRS-PRO	Age	−0.218	−0.375 to − 0.060	**0.007**	**0.121**

For NOSE-E and SNOT-22, removing sleep-related items did not materially alter the pattern of associations observed in the original models.

In contrast, for CRS-PRO, OSA was no longer retained as an independent predictor once sleep-related items were excluded. The only remaining predictor in the sleep-free CRS-PRO model was age, and the adjusted R² decreased to approximately 0.12.

### Summary of model performance

Across all analyses, the multivariable models demonstrated limited explanatory power, with modest adjusted R² values consistent with the exploratory nature of the study and the multifactorial determinants of CRS symptom burden. Diagnostics for model assumptions and variance inflation factors (Table 6) showed no violations of linear regression assumptions or collinearity concerns.

## Discussion

This study examined the influence of sociodemographic and clinical variables on symptom burden in patients with chronic rhinosinusitis without nasal polyps (CRSsNP) using three validated PROMs—NOSE-E, SNOT-22 and CRS-PRO—administered concurrently. Each instrument demonstrated a distinct pattern of associations, underscoring the heterogeneous nature of CRSsNP and the importance of considering domain structure when interpreting patient-reported outcomes. Although the explanatory capacity of our models was modest, this is consistent with the established multifactorial and subjective character of CRS symptom perception, which is known to depend on biological, psychosocial, and environmental determinants that extend beyond the routinely measured clinical variables [12–14].

### PROM-specific interpretation of findings

The NOSE-E, a targeted measure of nasal obstruction, exhibited a predictor profile distinct from the broader PROMs. Age was inversely associated with NOSE-E scores, suggesting decreased perceived obstruction among older adults. Similar age-related reductions in symptom reporting have been previously described and may reflect diminished sensory perception, altered cognitive appraisal of airflow limitation, or lower expectations regarding nasal function in later life [15, 16]. Smoking also remained independently associated with worse NOSE-E scores. This aligns with evidence that tobacco exposure induces chronic mucosal inflammation, reduces mucociliary clearance, and potentiates subjective nasal congestion [17, 18]. Together, these findings emphasize the need to account for demographic and environmental exposures when interpreting NOSE-E results.

In contrast, the SNOT-22—which captures a wide spectrum of physical, emotional, and sleep-related symptoms—identified previous nasal surgery as the sole independent predictor in the multivariable model. This finding is in line with studies showing that patients requiring revision or persistent management often report worse quality of life, even after surgical intervention [19, 20]. The persistence of elevated SNOT-22 scores may reflect underlying inflammatory endotypes, structural sequelae, or unresolved patient expectations. The absence of associations with other comorbidities highlights the instrument’s sensitivity to accumulated disease burden rather than discrete systemic factors.

The CRS-PRO revealed yet another dimension of symptom experience. In the primary analysis, both age and obstructive sleep apnea (OSA) were independently associated with CRS-PRO scores. The inverse association with age mirrors the patterns observed in the NOSE-E, reinforcing prior findings that older CRS patients frequently report lower symptom intensity [21, 22]. The independent association with OSA initially suggested that sleep-disordered breathing may contribute meaningfully to symptom burden in CRSsNP, a relationship supported by prior evidence linking CRS with sleep disruption and fatigue [4, 9, 23]. However, the interpretability of this association required further scrutiny given the presence of sleep-related items within the PROM structure itself.

### Influence of sleep-related item content on the OSA association

A major strength of this study was the preplanned sensitivity analysis that removed sleep- and fatigue-related items from all PROMs. This analysis directly addressed the risk of a tautological association between OSA and PROM scores, particularly because several items embedded in CRS-PRO and SNOT-22 reflect constructs such as poor sleep, tiredness, or daytime dysfunction—domains that are intrinsically affected by OSA.

Our findings revealed that once sleep-related items were removed, the association between OSA and CRS-PRO was no longer present. Age remained the only significant predictor, and the explained variance decreased accordingly. This strongly suggests that the initial OSA signal was driven predominantly by overlapping item content rather than by sinonasal disease burden itself. These observations are consistent with studies demonstrating that sleep quality is a major determinant of CRS-related quality of life [9, 23], and with conceptual reviews emphasizing that PROM interpretation must account for domain weighting, item content, and potential construct overlap [5, 19, 24].

Importantly, the removal of sleep-related items did not materially alter the predictor profiles of the NOSE-E or SNOT-22. This divergence likely reflects the minimal weighting of sleep-related content in NOSE-E and the diffuse, multi-domain structure of SNOT-22, where sleep items represent only a fraction of the total score. In CRS-PRO, however, sleep and fatigue domains comprise a more substantial portion of the instrument, making it more sensitive to sleep-related comorbidities such as OSA.

### Comparison with previous literature

The distinct behavior of the three PROMs in this study aligns with the broader literature on CRS phenotyping, which consistently demonstrates that symptom domains—not objective findings alone—shape quality-of-life outcomes [1–3]. The association between smoking and nasal obstruction is well established [25, 26], as is the tendency for patients with prior sinus surgery to exhibit persistently elevated SNOT-22 scores [20, 24]. The attenuation of the OSA signal following item removal adds novel methodological insight by showing how domain structure can influence the apparent relationship between comorbidities and patient-reported burden. This is especially relevant in CRSsNP, a phenotype where inflammatory drivers are often less pronounced than in CRSwNP, making subjective contributors more prominent.

### Strengths and limitations

This study has several strengths. The concurrent administration of three validated PROMs allowed direct comparison of their sensitivity to clinical predictors within the same cohort, providing valuable insight into domain-specific behavior. The sleep-free sensitivity analysis represents a meaningful methodological advance, enabling us to disentangle true clinical associations from item-level overlap. The use of real-world clinical data from a tertiary center adds ecological validity.

Several limitations must also be acknowledged. The sample size was modest, increasing the risk of overfitting and limiting statistical power. The low adjusted R² values indicate that much of the variance in PROM scores remains unexplained and likely reflects unmeasured psychosocial, inflammatory, or environmental factors [13, 14]. The tertiary setting may limit generalizability, as patients in specialty clinics often present with more complex or refractory disease. Comorbidities were extracted from medical records rather than standardized testing, raising the possibility of misclassification. Moreover, the cross-sectional design precludes causal inference.

### Clinical implications and future directions

These findings highlight the importance of selecting PROMs according to their domain structure and the specific clinical question at hand. In patients with suspected or established OSA, PROMs that heavily weight sleep-related items—such as CRS-PRO—should be interpreted cautiously, as scores may reflect sleep disturbance rather than sinonasal disease activity. Future studies should incorporate longitudinal assessments, objective sleep evaluations, and more granular inflammatory or psychosocial measures to better delineate predictors of symptom burden in CRSsNP.

## Conclusion

This study shows that sociodemographic and clinical factors exert heterogeneous and instrument-specific effects on patient-reported outcomes in CRSsNP. Age and smoking independently influenced nasal obstruction scores, previous nasal surgery was the only consistent predictor of global symptom burden on the SNOT-22, and both age and obstructive sleep apnea were associated with CRS-PRO scores in the main analysis. However, the planned sensitivity analysis demonstrated that the apparent relationship between OSA and CRS-PRO was largely mediated by sleep-related item content, as this association disappeared once these items were removed. These findings underscore the importance of interpreting PROMs in CRSsNP with awareness of their domain structure, particularly when sleep-related comorbidities are present.

Across all models, the modest adjusted R² values highlight the multifactorial and subjective nature of CRS symptom burden, and indicate that commonly measured clinical variables explain only a small proportion of patient-reported outcomes. Consequently, PROM-based research in CRSsNP should incorporate domain-specific interpretation, avoid overreliance on isolated predictors, and recognize the limitations inherent to exploratory modelling in small samples.

Future studies with larger and more diverse cohorts, longitudinal designs, and objective assessments of sleep and inflammatory status are needed to better delineate the mechanisms driving symptom perception in CRSsNP and to clarify the true contribution of comorbidities such as OSA beyond their overlap with sleep-related questionnaire content.

## Electronic Supplementary Material

Below is the link to the electronic supplementary material.

Supplementary Material 1

## Data Availability

The datasets generated and analyzed during the current study are not publicly available due to patient privacy restrictions but are available from the corresponding author on reasonable request.

## Abbreviations

CRS: Chronic Rhinosinusitis
CRSwNP: Chronic Rhinosinusitis with Nasal Polyps
CRSsNP: Chronic Rhinosinusitis without Nasal Polyps
PROM: Patient-Reported Outcome Measure
NOSE-E: Nasal Obstruction Symptom Evaluation – Spanish version
SNOT-22: Sino-Nasal Outcome Test–22
CRS-PRO: Chronic Rhinosinusitis Patient-Reported Outcome
OSA: Obstructive Sleep Apnea
BMI: Body Mass Index
SD: Standard Deviation
CI: Confidence Interval
VIF: Variance Inflation Factor
EPOS: European Position Paper on Rhinosinusitis and Nasal Polyps
