# Supplementary material for: Systemic predictors of symptom burden in CRSsNP: a cross-sectional comparison of three patient-reported outcome measures
Source: Eur Arch Otorhinolaryngol. 2026 Jan 20;283(3):1795–802. doi: 10.1007/s00405-026-10014-4 (PMC13002631; doi:10.1007/s00405-026-10014-4)
Supplement: Supplementary file 1 — Supplementary Material 1 [file 405_2026_10014_MOESM1_ESM.docx]

**SUPPLEMENTARY TABLE S1. Distribution of original and sleep-free PROM scores**

| **PROM** | **Mean** | **SD** | **Median** | **Range** |
| --- | --- | --- | --- | --- |
| NOSE-E | 11.34 | 4.53 | 12 | 0–20 |
| SNOT-22 | 45.18 | 23.38 | 44.5 | 1–91 |
| CRS-PRO | 20.60 | 10.22 | 21 | 1–48 |
| NOSE-E_sf | 9.08 | 3.58 | 10 | 0–16 |
| SNOT-22_sf | 37.06 | 18.85 | 37 | 1–72 |
| CRS-PRO_sf | 17.14 | 8.36 | 17 | 1–40 |
